# A Combined Randomised and Observational Study of Surgery for Fractures In the distal Radius in the Elderly (CROSSFIRE): a statistical analyses plan

**DOI:** 10.1186/s13063-020-4228-0

**Published:** 2020-07-15

**Authors:** Andrew Lawson, Justine Naylor, Rachelle Buchbinder, Rebecca Ivers, Zsolt Balogh, Paul Smith, Rajat Mittal, Wei Xuan, Kirsten Howard, Arezoo Vafa, Piers Yates, Bertram Rieger, Geoff Smith, Ilia Elkinson, Woosung Kim, Jai Sungaran, Kim Latendresse, James Wong, Sameer Viswanathan, Keith Landale, Herwig Drobetz, Phong Tran, Richard Page, Raphael Hau, Jonathan Mulford, Ian Incoll, Michael Kale, Bernard Schick, Andrew Higgs, Andrew Oppy, Diana Perriman, Ian Harris

**Affiliations:** 1 Whitlam Orthopaedic Research Centre, Ingham Institute for Applied Medical Research, Sydney, NSW Australia; 2 South Western Sydney Clinical School, UNSW, Sydney, NSW Australia; 3 Monash University, Melbourne, VIC Australia; 4 Cabrini Institute, Melbourne, VIC Australia; 5 School of Public Health and Community Medicine, UNSW Sydney, Sydney, NSW Australia; 6 John Hunter Hospital, Newcastle, NSW Australia; 7 Canberra Hospital, Canberra, ACT Australia; 8 Ingham Institute for Applied Medical Research, Sydney, NSW Australia; 9 Faculty of Medicine and Health, University of Sydney, Sydney, NSW Australia; 10 Fiona Stanley Hospital, Perth, WA Australia; 11 St George and Sutherland Hospitals, Sydney, NSW Australia; 12 Wellington Hospital, Wellington, New Zealand; 13 Concord Hospital, Sydney, NSW Australia; 14 Nambour Hospital and Sunshine Coast University Hospital, Nambour, QLD Australia; 15 Westmead Hospital, Sydney, NSW Australia; 16 Campbelltown Hospital, Sydney, NSW Australia; 17 Mackay Base Hospital, Mackay, QLD Australia; 18 Western Health, Melbourne, VIC Australia; 19 University Hospital Geelong, Barwon Health, Geelong, NSW Australia; 20 School of Medicine, Deakin University, Geelong, VIC Australia; 21 Northern Health, Melbourne, VIC Australia; 22 Launceston Hospital, Launceston, TAS Australia; 23 Gosford and Wyong Hospitals, Gosford, NSW Australia; 24 Prince of Wales Hospital, Sydney, NSW Australia; 25 St Vincent’s Hospital, Sydney, NSW Australia; 26 Royal Melbourne Hospital, Melbourne, VIC Australia; 27 Liverpool Hospital, Sydney, NSW Australia

**Keywords:** Aged, Fracture fixation, Plaster casts, Radius fractures, Randomised controlled trial, Recovery of function, Statistical analysis plan

## Abstract

**Background:**

We are performing a combined randomised and observational study comparing internal fixation to non-surgical management for common wrist fractures in older patients. This paper describes the statistical analysis plan.

**Methods/design:**

A Combined Randomised and Observational Study of Surgery for Fractures In the distal Radius in the Elderly (CROSSFIRE) is a randomised controlled trial comparing two types of usual care for treating wrist fractures in older patients, surgical fixation using volar locking plates and non-surgical treatment using closed reduction and plaster immobilisation. The primary aim of this comparative-effectiveness study is to determine whether surgery is superior to non-surgical treatment with respect to patient-reported wrist function at 12 months post treatment. The secondary outcomes include radiographic outcomes, complication rates and patient-reported outcomes including quality of life, pain, treatment success and cosmesis. Primary analysis will use a two-sample *t* test and an intention-to-treat analysis using the randomised arm of the study. Statistical analyses will be two-tailed and significance will be determined by *p* < 0.05. Sensitivity analyses will be conducted to assess for differences in intention-to-treat, per-protocol and as-treated analyses. Sensitivity analyses will also be conducted to assess selection bias by evaluating differences in participants between the randomised and observational study arms, and for bias relating to any missing data. An economic analysis will be conducted separately if surgery is shown to provide superior outcomes to a level of clinical significance.

**Discussion:**

This statistical analysis plan describes the analysis of the CROSSFIRE study which aims to provide evidence to aid clinical decision-making in the treatment of distal radius fractures in older patients.

**Trial registration:**

CROSSFIRE was approved by The Hunter New England Human Research Ethics Committee (HNEHREC Reference No: 16/02/17/3.04). Registered on 22 July 2016 with The Australian and New Zealand Clinical Trials Registry (ANZCTR Number; ACTRN12616000969460).

This manuscript is based on v.11 of the statistical analysis plan. A copy of v.11, signed by the chief investigator and the senior statistician is kept at the administering institution.

## Introduction

### Background

Fractures of the distal radius are the most common fractures presenting to emergency departments and orthopaedic surgeons [1]. These fractures are more common in older people (due to osteoporosis and increased risk of falls) and the incidence in older people is increasing [1]. Considerable practice variation exists in the management of distal radius fractures in the elderly in Australia [2], with two common methods being closed reduction (manipulation of the arm to realign the fracture) with cast immobilisation, and open reduction (surgical exposure and direct realignment of the fracture) and fixation with plate and screw. Open reduction and (volar locking) plate fixation has become the most common form of surgical treatment provided [3]. While there is evidence showing no significant advantage for some forms of surgical fixation over closed treatment, and no difference between different surgical techniques [4–16], there is a lack of evidence comparing the two most common treatments used in Australia, initial treatment with volar locking plate fixation versus closed reduction and cast immobilisation. Surgical management of these fractures involves significant costs (implant costs, medical costs, hospital costs) and risks (infection, implant failure, general surgical risks) compared to non-surgical management (closed reduction and cast immobilisation in the emergency department). Therefore, high-level evidence comparing the current treatment alternatives (plate fixation versus casting) is required in order to address practice variation, justify or avoid costs, and to provide the best clinical outcome for patients with these common fractures.

The CROSSFIRE study protocol was published in 2017 [17]. This statistical analysis plan (SAP) was prepared in accordance with published guidelines [18] on the content of SAPs and was written with input from members of The CROSSFIRE Study Group.

### Purpose of the analyses

The primary and secondary aims of the study are to determine whether surgical treatment (volar-locking-plate fixation) is superior to non-surgical treatment (closed reduction and cast immobilisation) with respect to both effectiveness and safety for adults aged 60 years and older with dorsally displaced distal radius fractures.

The results of the trial will be published in a peer-reviewed journal and will be disseminated via various forms of media. The results will be incorporated in clinical practice guidelines produced by professional bodies.

## Methods/design

### Design

CROSSFIRE is a prospective, multi-centre study with 19 sites in Australia and New Zealand. The study includes a randomised, comparative-effectiveness trial and a parallel observational study; eligible patients were invited to participate in the randomised trial in which their treatment was randomised to surgical or non-surgical treatment. Eligible patients who declined to participate in the randomised study were invited to join the observational study. Participants in the observational study received standard care (one of the two treatment arms in the randomised trial) according to patient and surgeon preference and had the same outcomes measured at the same timepoints as those participating in the randomised trial.

We included an observational ‘preference’ arm that followed non-randomised patients in a similar manner to randomised participants to investigate potential selection bias and provide information on the generalisability of the study [19]. This study design has been used in surgical trials [20] and has been recommended as a model for trials of surgery versus non-surgical treatment where recruitment rates are expected to be lower than for other randomised controlled trials (RCTs) [21].

### Study population

Three hundred participants provided consent and were recruited to CROSSFIRE from 19 participating hospital sites: 164 participants in the randomised trial and 136 in the observational study. No patients declined participation in the observational study.

### Inclusion criteria

- Age 60 years or older
- Displaced distal radius fracture, classified according to the Association for the Study of Internal Fixation/Orthopaedic Trauma Association (AO/OTA) [22] 23A (extra-articular distal radius fracture) or 23C (complete articular distal radius fracture). Displacement parameters include more than 10° dorsal angulation, referenced off a line perpendicular to the shaft of the radius or more than 3 mm shortening or more than a 2-mm articular step prior to reduction
- Medically fit for surgery
- Independent living (including hostel accommodation)
- Low-energy injury (fall from less than 1 m)
- Available for follow-up for 12 months

### Exclusion criteria

- Patient unable to provide consent (due to cognitive incapacity or lack of English proficiency)
- Volar angulation
- Diaphyseal extension
- Partial articular fractures (AO/OTA 23B)
- Associated fracture or dislocation in any other body part that will affect the use of the involved wrist (ulnar styloid fracture will be permitted, as these are usually associated with the fracture under investigation)
- Open injury
- Previous wrist fracture on the same side
- Medical condition precluding anaesthetic

### Intervention group

Surgical fixation using a volar locking plate was performed within 2 weeks of the initial injury according to the usual protocol of the participating institution, with an orthopaedic surgeon in attendance. This is a commonly performed procedure. Surgical technique and type of plate (make and length) was as per surgeon preference. A plaster cast was applied post-operatively but for no longer than 2 weeks. Active finger movement was encouraged post-operatively. Participants were reviewed 2 weeks (10–17 days) after surgery; the wound was reviewed and sutures removed where necessary. Participants were provided with a post-operative home-exercise programme (printed information). Referral for outpatient rehabilitation was not routinely provided but was permitted.

### Control group

Participants in this group were treated with a closed-reduction and cast immobilisation, avoiding wrist flexion, within 2 weeks of the initial injury. This method of casting is consistent with standard casting practice in Australia. Immobilisation of a distal radius fracture in flexion has been associated with an increased risk of fracture displacement as well as finger and metacarpophalangeal joint stiffness [23]. Also, immobilisation in a cast that is too restrictive and excessively flexed has been associated with an increased risk of complex regional pain syndrome (CRPS) [24, 25]. The reduction was performed in the emergency department under sedation and local anaesthetic infiltration into the fracture (haematoma block) where possible or in an operating room (if not possible in the emergency department). The procedure was performed by an orthopaedic surgeon or registrar. Post-reduction radiographs were taken to assess the fracture alignment. The best reduction achievable was accepted. The cast was removed at 6 (± − 1) weeks from the initial reduction. Active finger movement and light use of the hand was encouraged immediately. Participants were provided with a home-exercise programme (written information). Referral for outpatient rehabilitation was not routinely provided but was permitted.

### Patient and public involvement

The Consumer Advisory Group of ANZMUSC (the Australian and New Zealand Musculoskeletal Clinical Trials Network) reviewed the protocol and the study was endorsed by ANZMUSC. Separately, three elderly patients with wrist fracture (who were not study participants) were interviewed and provided feedback on what post-treatment information was most relevant and important to older patients with wrist fracture.

### Randomisation and allocation

Randomisation occurred immediately after consent was gained by the recruiting orthopaedic team, within 1 week of the date of the injury. The orthopaedic team member contacted a central computer-based randomisation service by telephone. Participants were randomised using the method of minimisation. Randomisation was stratified by site, and minimisation, adjusting for gender and age (60–74 years and > 74 years), was employed as recommended by the National Health and Medical Research Centre (NHMRC) Clinical Trials Centre which provided the randomisation service.

Given the nature of the comparisons (surgery versus no surgery), it was not possible to blind the surgeon (study) investigators or participants. While this increases the risk of performance and detection bias, every effort was made to ensure that treatment, other than the interventions under study was otherwise identical in both groups. The primary outcome (PRWE score at 12 months) was collected from participants by blinded researchers, by telephone.

The statistician and the investigators conducting the analysis will remain blinded to the treatment groups. The dataset will be de-identified and treatment allocation will be masked using dummy group names (for example, Group A and Group B). De-identification and masking will be performed by an independent investigator who is not associated with the analysis. Masking of treatment allocation will be maintained until the statistical analysis and interpretation has been completed and two versions of the manuscript have been prepared and agreed to by all authors.

### Data collection

Outcomes are collected at four timepoints; baseline, 3 months, 12 months and 24 months. The timepoints and corresponding outcomes are listed in Table 1. Baseline data is collected by the recruiting clinicians following consent. Baseline data includes age, gender, fracture type (AO/OTA 23A OR 23C), radiographic features of fracture, fracture-healing risk factors (see co-morbidities in Tables 2 and 3) and treatment preference. Paper-based data collection is forwarded to the study coordinator for direct electronic data entry into a central electronic database – REDCap™ [26].

**Table 1:** Study outcomes

Baseline measure		Randomised	
		Surgical (*n*=__)	Non-surgical (*n*=__)
Age (years), mean (range)			
Gender n (%)	Female		
	Male		
Fracture type n (%)	23A		
	23C		
Radiographic features, mean (SD)	Dorsal angulation (degrees)		
	Radial tilt (degrees)		
	Ulnar variance (mms)		
	Articular step (mms)		
Co-morbidities, n (%)	Diabetes? (Y/N)		
	Smoker? (Y/N)		
	Glucocorticoid treatment? (Y/N)		
	Osteoporosis treatment? (Y/N)

**Table 2:** Baseline characteristics for the randomised group

Baseline measure	Randomised	
	Surgical (*n* = __)	Non-surgical (*n* = __)
Age (years), mean (range)		
Gender *n* (%)		
Female		
Male		
Fracture type *n* (%)		
23A		
23C		
Radiographic features, mean (SD)		
• Dorsal angulation (degrees)		
• Radial tilt (degrees)		
• Ulnar variance (mm)		
• Articular step (mm)		
Co-morbidities, *n* (%)		
• Diabetes? (Y/N)		
• Smoker? (Y/N)		
• Glucocorticoid treatment? (Y/N)		
• Osteoporosis treatment? (Y/N)		

*SD* standard deviation

**Table 3:** Baseline characteristics for the observational group

Baseline measure		Observational	
		Surgical (*n*=__)	Non-surgical (*n*=__)
Age (years), mean (range)			
Gender n (%)	Female		
	Male		
Fracture type n (%)	23A		
	23C		
Radiographic features, mean (SD)	Dorsal angulation (degrees)		
	Radial tilt (degrees)		
	Ulnar variance (mms)		
	Articular step (mms)		
Co-morbidities, n (%)	Diabetes? (Y/N)		
	Smoker? (Y/N)		
	Glucocorticoid treatment? (Y/N)		
	Osteoporosis treatment? (Y/N)		

Participants are followed up at 3 months post initial procedure (± 1 week), 12 months post initial procedure (± 1 month) and 24 months post initial procedure (± 1 month). There is an intention to collect outcomes at 5 and 10 years post treatment.

Participant follow-up at 3-month, 12-month and 24-month timeframes is conducted by telephone. The outcomes for the randomised arm of the study are collected by a blinded investigator. On rare occasions where data collection by telephone is not practical or possible (hearing impairment, English language proficiency), paper questionnaires are sent by post or by email to the participant to be completed and sent back to the research institution by reply-paid post. Completed telephone and postal questionnaires are entered into the password-protected central electronic database and paper-based records are collected and securely stored within the administering institution.

### Outcome variables

The primary outcome is the patient-rated wrist evaluation (PRWE) questionnaire at 12 (± 1) months post injury. The PRWE is a 15-item patient-reported measure of pain and function, specific to the wrist. It is a continuous score on a scale from 0 to 100 with higher scores indicating poorer outcomes [27]. It is commonly used, was developed with patient input and has been validated for use in patients with distal radius fractures [28].

Secondary outcomes include:

- PRWE gathered at 3 months and 2, 5 and 10 years
- Disability of Arm, Shoulder and Hand (DASH) questionnaire at 12 months. The DASH is a 30-item patient-reported measure of disability and symptoms of the upper limb. It is a continuous score on a scale from 0 to 100 with higher scores indicating poorer outcomes [29]
- The EuroQol five-dimension five-level questionnaire (EQ-5D-5L), measuring health-related utility-based quality of life at 3 and 12 months and 2, 5 and 10 years. The EQ-5D-5L is a five-dimension patient-reported measure of health-related quality of life (HRQoL) with a separate visual analogue scale (VAS). Distributions of responses to each dimension can be reported categorically; it also produces two continuous variables, a EQ-utility index (EQ-UI) score and a EQ-VAS score. The EQ-UI score is scored on a scale of 0, equating to a health state equivalent to death and 1 equating to full health; it can also take negative values. Given that there are no published Australian normative data on which to calculate EQ-UI scores, UK norms will be used instead. The EQ-VAS score ranges from 0 to 100 with 0 equating to the worst health state imaginable and 100 equating to the best health state imaginable [30]
- Wrist pain using the numerical rating scale (NRS) at 3 and 12 months and 2, 5 and 10 years. The NRS is a patient-reported 11-point pain scale ranging from 0 to 10 with 0 equating to no pain and 10 equating to the worst possible pain
- Patient-reported treatment success (at 12 months and 2, 5 and 10 years, 5-point Likert scale). The treatment success scale is a non-standard scale. See Additional file 1
- Patient-rated bother with appearance (at 12 month and 2, 5 and 10 years, 5-point Likert scale). The treatment success scale is a non-standard scale. See Additional file 1
- Complications (including deep infection, reoperation, neuropathy, tendon irritation requiring treatment, tendon rupture, fracture non-union at 6 months, implant failure, complex regional pain syndrome, death) at 3 months, 12 months, 2, 5 and 10 years
- Radiographic measures (ulnar variance, dorsal angulation, radial tilt, articular step) measured at presentation, post reduction, and between 6 weeks and 12 months)
- Therapy utilisation up to 3 months (Yes/No) and continuing at 3 months (Yes/No)

### Adverse events

Adverse events are defined as symptomatic fracture non-union (three of four cortices not united radiographically at a minimum of 6 months), infection (local infection requiring any treatment), neuropathy, tendon irritation (requiring treatment), tendon rupture or complex regional pain syndrome (diagnosed according to the International Association for the Study of Pain (IASP) clinical diagnosis criteria [31]).

### Study hypotheses

The primary hypothesis is that patients aged 60 years and older with displaced fractures of the distal radius managed surgically using volar-locking-plate fixation will have a clinically important superior patient-rated pain and function (PRWE score) at 12 months post injury compared to those managed non-surgically with closed reduction and plaster casting. A difference of greater than 14 points will be considered to be clinically important [32].

The secondary hypothesis is that patients aged 60 years and older with displaced fractures of the distal radius managed surgically using volar-locking-plate fixation compared to those managed non-surgically with closed reduction and plaster casting, will have:

- Significantly lower rates of major complications (including deep infection, reoperation, neuropathy, tendon irritation requiring treatment, tendon rupture, fracture non-union, and implant failure, complex regional pain syndrome, death) at 3 and 12 months post treatment
- Superior (closer to normal) radiographic outcomes (ulnar variance and articular step (millimetres), dorsal angulation and radial tilt (degrees)) at post reduction and 6 weeks or more
- Superior PRWE scores at 3 months, 2, 5 and 10 years to a level of clinical importance
- Superior DASH scores at 12 months, to a level of clinical importance. A difference of greater than 11 points will be considered to be clinically important [33]
- Superior health-related quality of life (EQ-UI and EQ-VAS scores at 3 months and 12 months, 2, 5 and 10 years to a level of clinical importance. A difference of greater than 0.1 points in the EQ-UI score has previously been used as the minimal clinically important difference (MCID) in orthopaedic surgery [34] and musculoskeletal [35] contexts and will be considered to be clinically important. A difference of 10 on the EQ-VAS has been reported as the MCID and will be considered to be clinically important [36]
- Lower patient-reported pain (numerical rating scale NRS) at 3 months and 12 months, 2, 5 and 10 years to a level of clinical importance. A systematic review on the MCIDs of Visual Analogue Scales (VAS) used for assessment of acute pain reported that the median MCID was 17 mm on a 100-mm scale [37]. Given that the NRS uses whole integers between 0 and 10, a value of 2 points or greater will be considered to be clinically important
- Superior patient-reported treatment success at 12 months, 2, 5 and 10 years (measured on a 5-point Likert scale)
- Less patient-rated bother with appearance at 12 months, 2, 5 and 10 years (measured on a 5-point Likert scale)
- Lower therapy utilisation up to and at 3 months as measured by the prevalence of therapy utilisation at 3 months

### Power and sample size

We consider 14 points on PRWE to be the minimum clinical difference necessary to justify the additional costs of surgery compared to non-surgical treatment. This is the MCID determined by Sorensen [32] and larger than the 11.5-point estimate determined by Walenkamp [38].

A total of 128 patients (64 in each group) will provide 90% power to detect a difference of 14 points on the PRWE scale at a significance level of 0.05. We aim to recruit 160 patients to allow for 20% loss to follow-up. Two previous RCTs that had each published results at the time of our sample size calculation reported loss-to-follow-up rates of 19% [5, 39]. Given that this is a multi-centre trial, the closing date for recruitment (31 December 2018) was set based on expected recruitment of 160 randomised participants. However, by the time recruitment closed, 164 participants had been recruited.

The observational arm includes all eligible patients not consenting to randomisation. We recruited 136 participants to the observational arm to the trial.

### Statistical analysis

#### Analysis principles

Data will be analysed using statistical analysis package SAS (SAS Institute Inc., Cary, NC, USA) and R statistical computing software [40].

Recruitment for CROSSFIRE concluded in January 2019. The final analysis will commence after the 12-month data collection is complete. The de-identified data-set will be inspected for missing data and data quality.

Study outcomes produce both continuous and categorical data. Continuous data will be inspected for normality. Continuous data will be presented by mean values and standard deviations (SDs) as well as mean difference (MD) and 95% confidence interval (CIs) (see Tables 4 and 5 as examples) if normally distributed, otherwise by median and interquartile range.

**Table 4:** Secondary outcomes at 3 months for the randomised group

Outcome		Randomised		
		Surgical (*n*=__)	Non-surgical (*n*=__)	MD (95%CI) or odds ratio (95%CI)
PRWE, mean (SD)				
ED-5D-5L, mean (SD)	EQ-UI			
	EQ-VAS			
Pain on NRS (0-10)				
Patient-reported treatment success n(%)	Very successful			
	Successful			
	Neutral			
	Unsuccessful			
	Very unsuccessful			
Therapy utilisation up to 3 months (Yes/No) n(%)				
Therapy utilisation at 3 months (Yes/No) n(%)				
Radiographic measures, mean (SD)	Dorsal angulation (degrees)			
	Radial tilt (degrees)			
	Ulnar variance (mms)			
	Articular step (mms)

**Table 5:** Secondary outcomes at 3 months for the observational group

Outcome		Observational		
		Surgical (*n*=__)	Non-surgical (*n*=__)	MD (95%CI) or odds ratio (95%CI)
PRWE, mean (SD)				
ED-5D-5L, mean (SD)	EQ-UI			
	EQ-VAS			
Pain on NRS (0-10)				
Patient-reported treatment success n(%)	Very successful			
	Successful			
	Neutral			
	Unsuccessful			
	Very unsuccessful			
Therapy utilisation up to 3 months (Yes/No) n(%)				
Therapy utilisation at 3 months (Yes/No) n(%)				
Radiographic measures, mean (SD)	Dorsal angulation (degrees)			
	Radial tilt (degrees)			
	Ulnar variance (mms)			
	Articular step (mms)			

Categorical data will be summarised according to frequency (*n*) and incidence (%) (see Table 5). Comparison between treatment groups will be tested using chi-squared and odds ratio with 95% CI.

Comparisons between treatment groups will be tested at the two-sided, 5% significance level. *P* values will be reported to three decimal places if *P* > 0.001. *P* values < 0.001 will be reported as < 0.001. Means and SDs will be reported to one decimal place.

#### Data integrity

The study compares two treatments that comprise usual care. A Data Safety Monitoring Board (DSMB) was not required to be convened.

Interim analysis of data completeness and accuracy of data entry was conducted on two occasions during the data collection phase. The paper files from data collected by telephone were cross-checked against the electronic database. An audit of x-ray data was also be completed.

There are potential sources of bias. The types of bias and the efforts made to reduce their risk included:

- Selection bias; randomisation occurs immediately after consent and immediately prior to treatment assignment using a central computer-based randomisation service by telephone, using stratification and minimisation to ensure balanced treatment allocation for site, age and gender
- Performance bias; treatment, other than the interventions under study is identical in both groups. Participating surgeons have equipoise regarding the two treatment alternatives and were instructed not to express a treatment preference to the participants recruited to the randomised study
- Detection bias; the primary outcome is collected from participants by blinded researchers, by telephone. The statistician and investigators will be blinded to the treatment group during the analysis
- Attrition bias; attempts are made to minimise missing data, such as obtaining multiple contact details at recruitment and using telephone follow up rather than mail. Interim analyses of data completeness and accuracy of data entry are conducted on two occasions in the data collection period
- Reporting bias; the de-identified participant-level dataset and statistical code will be made available for collaborative research projects. All pre-specified outcomes will be reported

### Methods for handling missing data

In the analysis, missing data will be dealt with according to the instructions on the use of the outcome tools (PRWE, DASH and EQ-5D-5L). The need to impute will be assessed and confirmed at the time of review of blind data. If greater than 10% of the primary outcome data is missing from the randomised sample, then missing primary outcome data will be imputed using multiple imputations based on baseline data including age, gender and fracture type as well as PRWE score at 3 months. A completed case analysis will be performed as a sensitivity analysis.

### Trial profile (CONSORT flow) and baseline characteristics

**Fig. 1:**
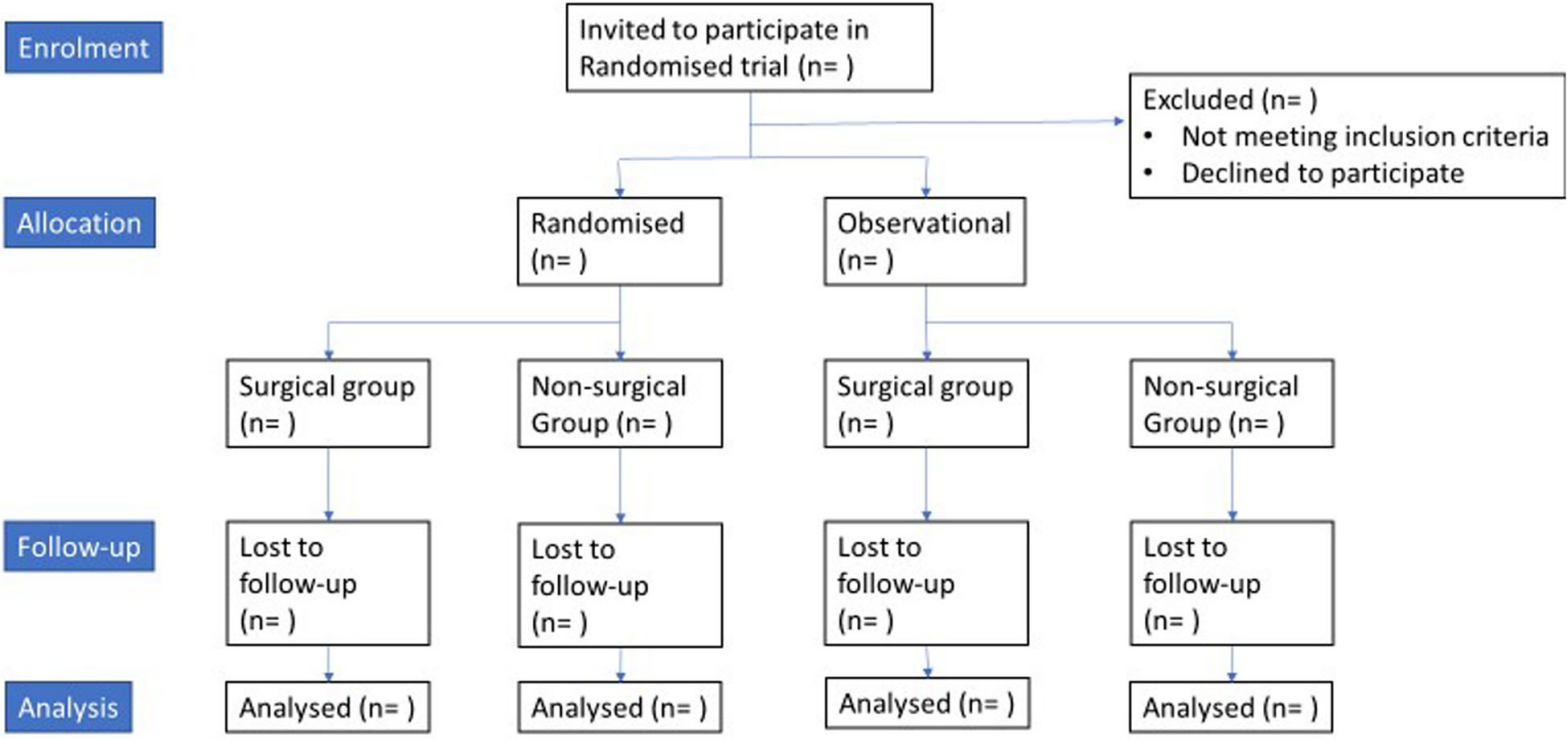
Participant flow

The flow of participants through the study will be displayed according to Consolidated Standards of Reporting Trials (CONSORT) (see Fig. 1 for example), including numbers of participants for enrolment, allocation, follow-up and analysis.

Participant characteristics will be displayed according to their baseline characteristics for each group. Characteristics will be displayed for both the randomised and the observational arms of the study and displayed for ease of comparison (see Tables 2 and 3 for examples).

### Primary analysis

**Fig. 2:**
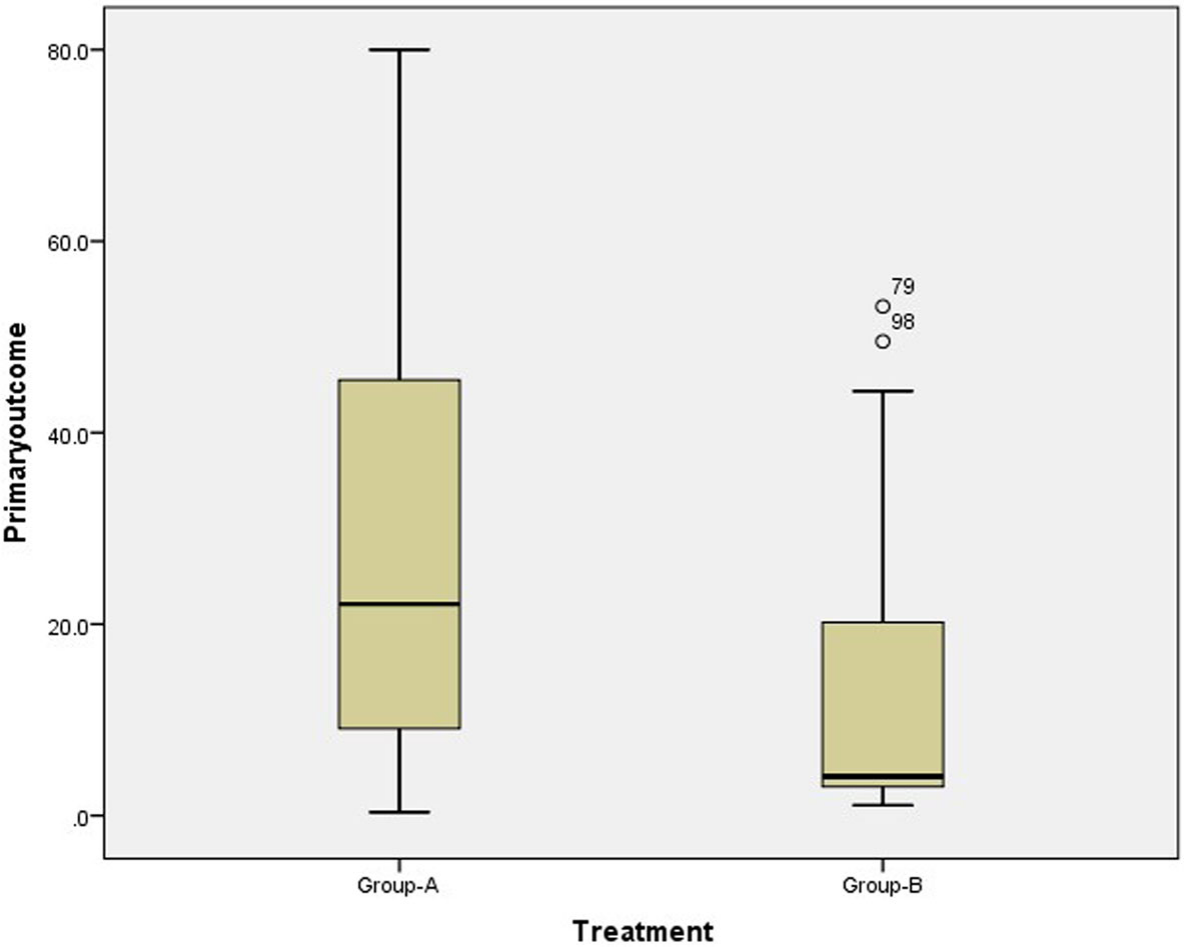
Box plot of primary outcome

The primary analysis will follow the intention-to-treat (ITT) principle. The primary outcome is the PRWE score at 12 months for the participants recruited to the randomised trial. The MD in PRWE scores between the surgical and non-surgical groups will be tested using a two-sample *t* test. The mean values, SDs, MDs and 95% CIs will be displayed (see Table 6 for example). The medians and inter-quartile ranges will be represented in a box plot (see Fig. 2 for example).

**Table 6:** Primary and secondary outcomes at 12 months for the randomised group

Outcome		Randomised		
		Surgical (*n*=__)	Non-surgical (*n*=__)	Mean difference (95%CI)
PRWE				
DASH				
ED-5D-5D, mean (SD)	EQ-UI			
	EQ-VAS			
Pain on NRS (0-10)				
Patient-reported treatment success n(%)	Very successful			
	Successful			
	Neutral			
	Unsuccessful			
	Very unsuccessful			
Patient-reported bother with appearance n(%)	Not at all			
	Bothered a little			
	Bothered moderately			
	Very bothered			
	Extremely bothered			
Radiographic measures, mean (SD)	Dorsal angulation (degrees)			
	Radial tilt (degrees)			
	Ulnar variance (mms)			
	Articular step (mms)

### Secondary analysis

Outcomes for the randomised group and the observational group will be analysed and represented separately. Outcomes gathered at 3 months will be displayed for the randomised group (see Table 4) and for the observation group (see Table 5). Outcomes gathered at 12 months will be displayed for the randomised group (see Table 6) and for the observational group (see Table 7). Continuous outcomes will be reported by mean values as well as the MDs and 95% CIs. Categorical outcomes will be reported by frequency, incidence and odds ratios.

**Table 7:** Primary and secondary outcomes at 12 months for the observational group

Outcome		Observational		
		Surgical (*n*=__)	Non-surgical (*n*=__)	Mean difference (95%CI)
PRWE				
DASH				
ED-5D-5D, mean (SD)	EQ-UI			
	EQ-VAS			
Pain on NRS (0-10)				
Patient-reported treatment success n(%)	Very successful			
	Successful			
	Neutral			
	Unsuccessful			
	Very unsuccessful			
Patient-reported bother with appearance n(%)	Not at all			
	Bothered a little			
	Bothered moderately			
	Very bothered			
	Extremely bothered			
Radiographic measures, mean (SD)	Dorsal angulation (degrees)			
	Radial tilt (degrees)			
	Ulnar variance (mms)			
	Articular step (mms)			

The MDs of PRWE measured at 3 months between the surgical and non-surgical groups will be tested using two-sample *t* tests. The MDs of DASH measured at 12 months between the surgical and non-surgical groups will be tested using two-sample *t* tests.

Physical therapy utilisation up to and at 3 months will be analysed. If surgical treatment is shown to be superior, a cost-effectiveness analysis will be conducted incorporating therapy utilisation and published separately to this analysis. For the purpose of this analysis, therapy utilisation up to 3 months (Yes/No) and ongoing therapy utilisation at 3 months (Yes/No) will each be reported in terms of frequency, incidence and odds ratio between each treatment group. See Tables 3 and 4 as examples.

Complications will be reported in terms of frequency, incidence and the odds ratio between each treatment group (see Tables 8 and 9 as examples). Odds ratio will be calculated based on the number of participants for whom data is available.

**Table 8:** Complications for randomised group

Complication; frequency (incidence)	Randomised group		
	Surgical (*n* = _)	Non-surgical (*n* = _)	Odds ratio (95% CI)
Deep infection	*n* (%)	*n* (%)	
Reoperation	*n* (%)	*n* (%)	
Neuropathy	*n* (%)	*n* (%)	
Tendon irritation requiring treatment	*n* (%)	*n* (%)	
Tendon rupture	*n* (%)	*n* (%)	
Fracture non-union at 6 months	*n* (%)	*n* (%)	
Implant failure	*n* (%)	*n* (%)	
CRPS	*n* (%)	*n* (%)	
Death	*n* (%)	*n* (%)	
Any complications	*n* (%)	*n* (%)	

*CI* confidence interval, *CRPS* chronic regional pain syndrome

**Table 9:** Complications for observational group

Complication; frequency (incidence)	Randomised group		
	Surgical (*n* = _)	Non-surgical (*n* = _)	Odds ratio (95% CI)
Deep infection	*n* (%)	*n* (%)	
Reoperation	*n* (%)	*n* (%)	
Neuropathy	*n* (%)	*n* (%)	
Tendon irritation requiring treatment	*n* (%)	*n* (%)	
Tendon rupture	*n* (%)	*n* (%)	
Fracture non-union at 6 months	*n* (%)	*n* (%)	
Implant failure	*n* (%)	*n* (%)	
CRPS	*n* (%)	*n* (%)	
Death	*n* (%)	*n* (%)	
Any complications	*n* (%)	*n* (%)	

*CI* confidence interval, *CRPS* chronic regional pain syndrome

Repeated measures analysis will be used for the following:

- Continuous variables measured at 3 months and 12 months include EQ-5D-5L (UI and VAS scores), pain on NRS (0–10) and radiographic measures (dorsal angulation, radial tilt, ulnar variance and articular step). These measurements will be analysed using analysis of variance (ANOVA) with repeated measures
- Categorical variables measured at 3 months and 12 months include patient-reported treatment success (Likert) and patient-reported bother with appearance (Likert). See Additional file 1 for question format. These variables will be analysed by contingency table to compare the frequency of treatment success categories between the two randomised groups. Results will be displayed as odds ratio with 95% CIs (see Table 4 for example)

The observational group will be used to investigate potential selection bias. Baseline characteristics will be compared between the observational group and the randomised group (see Table 10 for example).

**Table 10:** baseline characteristics for the randomised and observational arms of trial

Baseline measure		Randomised arm (*n*=__)	Observational arm (*n*=__)
Age (years), mean (range)			
Gender n (%)	Female		
	Male		
Fracture type n (%)	23A		
	23C		
Radiographic features, mean (SD)	Dorsal angulation (degrees)		
	Radial tilt (degrees)		
	Ulnar variance (mms)		
	Articular step (mms)		
Co-morbidities, n (%)	Diabetes? (Y/N)		
	Smoker? (Y/N)		
	Glucocorticoid treatment? (Y/N)		
	Osteoporosis treatment? (Y/N)		

The 2-, 5- and 10-year outcomes will be analysed separately to this analysis and will be published at a later date.

### Sensitivity analyses

In order to investigate the impact of missing data, a completed case analysis of the primary and secondary outcomes at 12 months will be performed and compared with the analysis using imputed data (if required); see Table 11 for example.

**Table 11:** Sensitivity analysis. Primary and secondary outcomes at 12 months, based on completed cases analysis of randomised group

Outcome		Completed cases in randomised group		
		Surgical (*n*=__)	Non-surgical (*n*=__)	Mean difference (95%CI)
PRWE				
DASH				
ED-5D-5D, mean (SD)	EQ-UI			
	EQ-VAS			
Pain on NRS (0-10)				
Patient-reported treatment success n(%)	Very successful			
	Successful			
	Neutral			
	Unsuccessful			
	Very unsuccessful			
Patient-reported bother with appearance n(%)	Not at all			
	Bothered a little			
	Bothered moderately			
	Very bothered			
	Extremely bothered			
Radiographic measures, mean (SD)	Dorsal angulation (degrees)			
	Radial tilt (degrees)			
	Ulnar variance (mms)			
	Articular step (mms)			

In order to investigate the impact of non-compliance with the protocol, an as-treated analysis and a per-protocol analysis of the primary and secondary outcomes at 12 months will be performed and the results will be compared with the ITT analysis; see Tables 12 and 13 as examples. Non-operative treatment will be defined as a minimum of 28 days in the plaster splint for the purposes of the per-protocol analysis.

**Table 12:** Sensitivity analysis. Primary and secondary outcomes at 12 months, based on as-treated analysis of randomised group

Outcome		As-treated allocation of randomised group		
		Surgical (*n*=__)	Non-surgical (*n*=__)	Mean difference (95%CI)
PRWE				
DASH				
ED-5D-5D, mean (SD)	EQ-UI			
	EQ-VAS			
Pain on NRS (0-10)				
Patient-reported treatment success n(%)	Very successful			
	Successful			
	Neutral			
	Unsuccessful			
	Very unsuccessful			
Patient-reported bother with appearance n(%)	Not at all			
	Bothered a little			
	Bothered moderately			
	Very bothered			
	Extremely bothered			
Radiographic measures, mean (SD)	Dorsal angulation (degrees)			
	Radial tilt (degrees)			
	Ulnar variance (mms)			
	Articular step (mms)

**Table 13:** Sensitivity analysis. Primary and secondary outcomes at 12 months, based on per-protocol analysis of randomised group

Outcome		Per-protocol allocation of randomised group		
		Surgical (*n*=__)	Non-surgical (*n*=__)	Mean difference (95%CI)
PRWE				
DASH				
ED-5D-5D, mean (SD)	EQ-UI			
	EQ-VAS			
Pain on NRS (0-10)				
Patient-reported treatment success n(%)	Very successful			
	Successful			
	Neutral			
	Unsuccessful			
	Very unsuccessful			
Patient-reported bother with appearance n(%)	Not at all			
	Bothered a little			
	Bothered moderately			
	Very bothered			
	Extremely bothered			
Radiographic measures, mean (SD)	Dorsal angulation (degrees)			
	Radial tilt (degrees)			
	Ulnar variance (mms)			
	Articular step (mms)			

## Discussion

This plan describes the statistical analysis for CROSSFIRE. The primary analysis will determine whether surgery is superior to non-surgical treatment with respect to patient-reported wrist pain and function at 12 months. The secondary analyses will determine between-treatment differences at 3- and 12-month timepoints of outcomes including treatment complications and adverse events, patient-reported pain and function, quality of life, therapy utilisation, patient-reported treatment success, patient-reported bother with appearance and radiological outcomes. A sensitivity analysis will be conducted for potential biases, including a comparison between the observation and randomised arms of the study.

## Trial status

Participant recruitment was completed in January 2019 and collection of outcomes to 12 months post treatment is expected to be completed in December 2019. Data collection for 2-, 5- and 10-year outcomes will continue and will be analysed separately at a later date.

## Supplementary information

**Additional file 1.** Nonstandard outcome measures.

## Data Availability

A de-identified participant-level dataset and statistical code will be made available for collaborative research projects, on request of the chief investigator.

## Abbreviations

AO/OTA: Arbeitsgemeinschaft für Osteosynthesefragen/Orthopaedic Trauma Association
ANOVA: Analysis of variance
CI: Confidence interval
CRPS: Chronic regional pain syndrome
CROSSFIRE: A Combined Randomised and Observational Study of Surgery for Fractures In the distal Radius in the Elderly
DASH: Disabilities of the Arm, Shoulder and Hand
EQ-5D-5L: EQ-5D-5L; EuroQol instrument for assessing health status
ITT: Intention to treat
MCID: Minimal clinically important difference
MD: Mean difference
NHMRC: National Health and Medical Research Centre
NRS: Numerical Rating Scale
PRWE: Patient-rated wrist evaluation
RCT: Randomised controlled trial
SD: Standard deviation
VAS: Visual Analogue Scale
