# Supplementary material for: A Combined Randomised and Observational Study of Surgery for Fractures In the distal Radius in the Elderly (CROSSFIRE): a statistical analyses plan
Source: Trials. 2020 Jul 15;21:651. doi: 10.1186/s13063-020-4228-0 (PMC7364640; doi:10.1186/s13063-020-4228-0)
Supplement: Supplementary file 1 — Additional file 1. Nonstandard outcome measures. [file 13063_2020_4228_MOESM1_ESM.docx]

**Appendix 1; Nonstandard outcome measures**

- Patient-reported treatment success is a patient-reported expression of satisfaction with treatment. It produces a categorical variable on a Likert scale, ranging from *very successful* to *very unsuccessful*.

| 1. (Patient reported treatment success:) How successful was the treatment of your wrist fracture? | | | | |
| --- | --- | --- | --- | --- |
| Very successful | Successful | Neutral | Unsuccessful | Very unsuccessful |

- Bother is a patient-reported expression of how much the participant is bothered by the appearance of their wrist. It produces a categorical variable on a Likert scale, ranging from *not at all* to *extremely bothered*.

| 1. (Bother:) How much are you bothered by the appearance of your wrist? | | | | |
| --- | --- | --- | --- | --- |
| Not at All | Bothered A Little | Bothered Moderately | Very Bothered | Extremely Bothered |
